# Identifying C1orf122 as a potential HCC exacerbated biomarker dependently of SRPK1 regulates PI3K/AKT/GSK3β signaling pathway

**DOI:** 10.1016/j.gendis.2025.101721

**Published:** 2025-06-18

**Authors:** Jing Cai, Li Rong, Runzhi Wang, Zaikuan Zhang, Haiming Sun, Juan Chen, Dunchu Weng, Xinyi Li, Xiaosong Feng, Peiyi Lin, Shengming Xu, Zhihong Jiang, Yajun Xie, Qin Zhou

**Affiliations:** a Department of Biochemistry, School of Basic Medical Sciences, Harbin Medical University, Harbin, Heilongjiang 150081, China; b Department of Gastroenterology, Bishan Hospital of Chongqing Medical University, Chongqing 404000, China; c The Ministry of Education Key Laboratory of Laboratory Medical Diagnostics, The College of Laboratory Medicine, Chongqing Medical University, Chongqing 400016, China; d Department of Pathology, Bishan Hospital of Chongqing Medical University, Chongqing 404000, China

**Keywords:** C1orf122, Hepatocellular carcinoma, Phosphorylation, PI3K/AKT/GSK3β, SRPK1

## Abstract

Although Chromosome 1 open reading frame 122 (C1orf122) is known to be a protein-coding gene, its biological functions and mechanisms in hepatocellular carcinoma (HCC) remain unknown. Herein, bioinformatics analysis and experimental validation revealed that, C1orf122 was overexpressed in HCC tissues and cells, and correlated strongly with a poor prognosis of HCC patients. Subsequently, we knocked down and overexpressed C1orf122 in HCC cells, confirmed that C1orf122 significantly stimulated HCC cell growth and proliferation. Furthermore, flow cytometry and WB detection confirmed that C1orf122 significantly suppressed HCC cell apoptosis. Transwell migration and wound healing assays, along with WB analysis showed that C1orf122 strongly improved HCC cell migratory capacity. Mass spectrometry (MS) and Co-Immunoprecipitation (Co-IP) assays identified serine/arginine-rich protein-specific kinase 1 (SRPK1) as a C1orf122-interacting protein. Moreover, C1orf122 significantly upregulated total SRPK1 levels and suppressed SRPK1 protein phosphorylation at the Thr601 site. Using online prediction tools, we found that mTOR was the kinase of SRPK1 phosphorylating it at the Thr601 site, and other experiments confirmed that C1orf122 mediated SRPK1 Thr601 phosphorylation in a mTOR kinase-dependent manner. The cell phenotype assays further revealed that SRPK1 strongly stimulated the PI3K/AKT/GSK3β signaling pathway to enhance cell growth and migration. It was also observed that C1orf122 significantly activated the PI3K/AKT/GSK3β signaling pathway via SRPK1. To the best of our knowledge, this is the first study to demonstrate the involvement of the C1orf122-SRPK1-PI3K/AKT/GSK3β axis in HCC growth.

## Introduction

Primary liver cancer is a common malignant tumor worldwide, exhibiting a growing incidence. It is estimated that more than 850,000 new cases of primary liver cancer are diagnosed each year globally, making it the third leading cause of cancer-related deaths.^1^ Currently, primary liver cancer is classified into hepatocellular carcinoma (HCC), intrahepatic cholangiocarcinoma (ICC), and mixed-type liver cancer (HCC-ICC).^2^ Among these, HCC is the most common type, accounting for approximately 90% of all liver cancer cases, with highly heterogeneous pathological features and clinical manifestations.^3^ Research has shown that the development of HCC is a multi-factorial and multi-stage process, driven by abnormal activation of multiple signaling pathways.^4^ The occurrence of HCC is primarily influenced by chronic infections, with hepatitis B virus and hepatitis C virus being the major etiological factors.^5^ These factors can potentially induce post-hepatitis cirrhosis, thereby increasing the risk of developing HCC. Moreover, factors such as metabolic fatty liver^6^ and alcoholic liver disease^7^ are also recognized as high-risk factors for HCC development. Although treatment methods for HCC are increasingly being developed, its complex molecular mechanisms suggest that single therapeutic approaches cannot effectively control tumor progression. Therefore, it is imperative to explore the molecular pathogenic mechanisms of HCC and identify potential biomarkers and therapeutic targets.

In the molecular mechanism studies of HCC, Chromosome 1 open reading frame 122 (C1orf122), an emerging protein-coding gene, has attracted widespread attention. Although an analysis of The Cancer Genome Atlas (TCGA) database revealed that C1orf122 is abnormally overexpressed in various cancer types, especially in HCC, non-small cell lung cancer, breast cancer, and other tumors, the specific biological function and mechanism of C1orf122 is not fully known. Bioinformatics analyses have uncovered that C1orf122 overexpression is correlated with HCC development and predicts poor patient prognosis, suggesting that C1orf122 may play an important role in the pathogenesis of HCC. Further investigation is required to explore the biological functions and mechanisms of C1orf122 in HCC progression, especially its interaction with HCC-related signaling pathways.

Serine-arginine-rich protein kinase 1 (SRPK1), a kinase that specifically regulates the phosphorylation of serine/arginine-rich splicing factors, is markedly overexpressed in various cancers. This enzyme modifies the phosphorylation state of specific domains, influencing the selective splicing process of pre-mRNA, and its overactivation is closely associated with the pathological progression of several solid tumors, including HCC,^8^ non-small cell lung cancer,^9^ and gastric cancer.^10^ Mechanistically, abnormal expression of SRPK1 regulates multiple signaling pathways, such as the PI3K/AKT,^11^ transforming growth factor-beta (TGF-β),^12^ and mitogen-activated protein kinase (MAPK)^13^ pathways in different tumor types. The primary objective of this study was to investigate the role of C1orf122 in HCC and its underlying mechanisms. The results of this study showed that C1orf122 interacted with SRPK1 and regulated the phosphorylation level of SRPK1 at Thr601 via the mammalian target of Rapamycin (mTOR) kinase. Furthermore, C1orf122 activated the PI3K/AKT/GSK3β signaling pathway in an SRPK1-dependent manner, promoting HCC progression. As an important novelty aspect, this is the first study to identify the C1orf122's role in HCC via the SRPK1/PI3K/AKT/GSK3β signaling pathway. Our findings provide new insights into the molecular mechanisms of HCC and reveal potential targets for developing new therapeutic strategies for HCC. In summary, our results show that C1orf122 may be a promising biomarker for the early diagnosis and prognosis assessment of HCC. Future studies are advocated to clarify the role of C1orf122 in HCC and determine its therapeutic potential.

## Material and methods

### Antibodies

The following antibodies were used for Western blotting tests: C1orf122 (1:200, GeneTex, GTX45522), β-Tubulin (1:1000, Cell Signaling Technology, 2146S), Bax (1:1000, Cell Signaling Technology, 2772T), Bcl-2 (1:2000, Proteintech, 12789-1-AP), Phospho-Bcl-2 (1:2000, Proteintech, 80771-2-RR), Caspase-3 (1:1000, Cell Signaling Technology, 9662S), Cleaved-Caspase-3 (1:1000, Cell Signaling Technology, 9661T), E-Cadherin (1:1000, Cell Signaling Technology, 14472T), N-Cadherin (1:1000, Cell Signaling Technology, 13116T), Vimentin (1:1000, Invitrogen, MA5-11883), Slug (1:5000, Proteintech, 12129-1-AP), Twist1 (1:1000, Proteintech, 25465-1-AP), FLAG (1:1000, Invitrogen, MA1-91878), GFP (1:2000, Invitrogen, MA5-15256), PI3K (1:1000, Proteintech, 20584-1-AP), p-AKT (1:1000, Cell Signaling Technology, 9271T), AKT (1:1000, Cell Signaling Technology, 9272S), GSK3-β (1:1000, Cell Signaling Technology, 12456T), p-GSK3-β (1:1000, Cell Signaling Technology, 5558T), β-catenin (1:5000, Proteintech, 51067-2-AP), p-β-catenin (1:1000, Invitrogen, PA5-17685), P21 (1:500, MCE, HY-P80774), SRPK1 (1:1000, Cell Signaling Technology, 52012S) and p-SRPK1 (Thr601) (1:1000, Invitrogen, PA5-106156).

### Collection of HCC tissues

Twelve pairs of HCC tissues and surrounding tissues were collected from Bishan Hospital of Chongqing Medical University from 2023 to 2024. Seven pairs of tissues were snap-frozen utilizing nitrogen atmosphere and preserved at −80 °C, which were subsequently homogenized and used for protein extraction. The remaining five pairs of tissues were fixed with 4% paraformaldehyde (PFA) and stained with Crystal Violet Staining Reagent (Beyotime, C0121, China).

### Cell culture

The HuH-7, HepG2, Hep3B, SMMC7721, MHCC-97H, THLE-2, HeLa, and 293T cell lines were purchased from the ATCC cell bank and cultured at 37 °C under the conditions of 5% CO_2_ with the Dulbecco's Modified Eagle Medium (DMEM, Gibco, 11995065, United States) containing 10% Fetal Bovine Serum (FBS, Hyclone, SH30070, United States) and 1% Penicillin-Streptomycin Solution (Hyclone, SV30010, United States).

### RNA interference

si-C1orf122-sense: GCGAGGAGAUGUUACGGCA(dT) (dT), si-C1orf122-antisense: UGCCGUAACAUCUCUCCUCGC(dT) (dT). si-SRPK1-sense: GGACAAAGCCCAAAGGAAA(dT) (dT), si-SRPK1-antisense: UUUCCUUUGGGCUUUGUCC(dT) (dT). The si-RNA sequences were designed and synthesized by Tsingke. The cells were used for transfection experiments when they reached a density of about 60%. Lipofectamine 2000 Transfection Reagent (Invitrogen, 11668027, United states) was utilized to facilitate cell transfection. The ratio of siRNAs to Lipofectamine 2000 was 1:2, and the subsequent test was conducted 48 h post-transfection.

### RNA extraction and qRT-PCR

After 48 h of transfection, the cells were collected and homogenized to extract total RNA using the RNA Extraction Kit (Bioflux, BSC63S1, China). The extracted RNA was stored at −80 °C until use. Subsequently, the total RNA was reverse transcribed into cDNA using the PrimeScript™ RT reagent Kit (Perfect Real Time, TaKaRa, RR037A, Japan) and the mRNA levels of the target genes were quantified by Real Time PCR employing the TB Green® Premix Ex Taq™ (Tli RNaseH Plus, TaKaRa, RR420A, Japan).

### Plasmid construction

Primers targeting the genes of interest were designed and PCR amplification was performed to generate and verify the expected target fragments. The amplified products were obtained through agarose gels and inserted into either the CMV-GFP or p3 × FLAG vectors using ligation-independent cloning (LIC) following instructions on the respective kits. The recombinant plasmids were transformed into *E. coli* DH5α chemically competent cells via the heat-shock method. Briefly, 1 μL of plasmid DNA was added to 100 μL of competent cells, incubated on ice for 30 min, heat-shocked at 42 °C for 45 s, and then returned to the ice for 5 min. Subsequently, 500 μL of LB medium was added and the mixture was incubated at 37 °C while shaking for 1 h. The culture was plated onto LB agar containing appropriate antibiotics. Colonies were screened using the colony PCR method to identify positive clones, which were subsequently verified through Sanger sequencing. The verified strains were cultured in LB medium, and plasmids were extracted using the EndoFree Plasmid Midi Kit (CW Bio, CW2105S, China) following the manufacturer's instructions. The purified plasmids were stored at −20 °C for subsequent use.

### Western blot

Cells were transfected for 48 h and the culture media were removed. The cells were lysed by treatment with SDS and the protein concentration was determined using the BCA Protein Assay Kit (Thermo Fisher Scientific, YJ375835, United States). The proteins samples were mixed with the loading buffer and subjected to gel electrophoresis at 80 V. Subsequently, the proteins were transferred onto a PVDF membrane (Merck Millipore, IPVH00010, United states) followed by blocking in 5% nonfat dry milk (CST, 9999S, United States) for 2 h at room temperature. Subsequently, the membrane was incubated with primary antibody overnight at 4 °C. The membrane was washed with TBST 3 times, for 10 min each time, followed by incubation with secondary antibody at room temperature for 1 h. It was further washed with TBST and developed to visualize the protein bands.

### Establishment of a tumor xenograft model

Nude mice, aged 4–6 weeks, were selected for the study. A HepG2 cell line targeting C1orf122 was generated using CRISPR-Cas9 gene editing. Cells were harvested at approximately 90% confluence, counted, and resuspended. Nude mice were inoculated subcutaneously in the axillary region with approximately 2 million cells in a volume of 0.1 mL. The injection was administered slowly and gently. After the injection, the needle was gradually retracted, and a bulge developed at the injected site. The subcutaneous tumor volume was measured and the nude mice were weighed every four days after injection. At the 48th day, the nude mice were photographed at the tumor site, the tumors were excised, weighed and counted. All experimental animal procedures were approved by the Ethics Committee of Chongqing Medical University in China (approval number. IACUC-CQMU-2024-0102).

### Co-IP

At 48 h after transfection, the medium was removed and the cells were transferred into a tube containing the RM buffer for complete lysing under sterilize conditions. A specific amount of the cell lysate was mixed with the BCA reagent to determine the protein concentration. The RM buffer was added to equilibrate the solution together with 20 μL of Anti-GFP magnetic beads (Biolinkedin, L-1016, China). The mixture was mixed by rotating in a shaker at 4 °C overnight. After a 2-min incubation on a magnetic rack, the supernatant was carefully removed without disturbing the magnetic beads. Fresh RNA lysis buffer was then added to the beads. This washing step was repeated four times, with RLT buffer being replaced each time. Finally, 2x loading dye was added to the beads, and the sample was heated at 95 °C for 10 min. Subsequently, the magnetic beads were removed, and the eluted loading buffer was subjected to WB analysis, to determine the expression of target proteins.

### Immunohistochemistry

The tumor sections were boiled overnight in a 37 °C oven, dewaxed and rehydrated. They were then rinsed twice using ddH_2_O, followed by a repeat wash with PBS and antigen repair. Following antigen retrieval, the wells were permeabilized with Triton X-100 (Sigma–Aldrich, T8787, United States) and blocked with goat serum for 1 h at room temperature. Primary antibody was then added and incubated overnight at 4 °C. After three washes with TBST, the secondary antibody was incubated for 20 min at room temperature. The wells were then washed three times with TBST. Color development was performed using the DAB Horseradish Peroxidase Color Development Kit (Beyotime, P0203, China), followed by dehydration and blocking to be observed under an inverted microscope.

### CCK-8 assay

Cells were inoculated into 50 wells, whereas blank wells were filled with PBS to mitigate medium evaporation. The cultures were incubated at 37 °C in a 5% CO_2_ atmosphere. Each day, 10 μL of CCK-8 solution (MCE, HY-K0301, United States) was carefully added to each well, avoiding the introduction of air bubbles. After a 1-h incubation period, the optical density at 450 nm was measured. The resulting data were quantified and analyzed.

### Cloning formation

Cells growing at the logarithmic phase were collected and suspended in a medium for seeding into 6-well plates, with 500 cells per well. The cells were incubated at 37 °C in a 5% CO_2_ and monitored regularly. After approximately 2 weeks, cell clones could be visualized by the naked eye, and cells were collected and fixed with 4% PFA for 10 min. This was followed by staining with crystal violet for 10–20 min. The staining solution was washed and the cells were allowed to dry in the open air and photographs were taken for statistical analysis.

### Transwell migration

Cells growing at a logarithmic growth phase were placed into the cell chamber in a 24-well plate. About 200 μL of the cell suspension was added to the upper layer of the chamber, with 20,000 cells per well. To the lower layer, 0.5 mL of the medium without Penicillin-Streptomycin and serum was added in 24-well plates. During this process, care was taken to avoid air bubbles between the upper and lower layers of cells. The culture was then incubated for 48 h at 37 °C. Subsequently, the medium was removed, and the lower layer of the chamber was fixed with 4% PFA for 10 min. The cells were then stained with crystal violet for approximately 20 min and washed to remove excess stain. Images were captured using an inverted microscope and cell counts were performed. The bottom layer of the chamber was fixed with 4% PFA for 10 min. The lower layer was dyed for 20 min using crystal violet dye. The dye was removed and the cells were imaged using an inverted microscope and counted.

### Wound healing

Cells were cultured to reach a confluence of 90% in 6-well plates after which a 10 μL tip was used to create scratches on cell surface. The cell plate was gently and slow washed to remove unattached cells with PBS followed by addition of Penicillin-Streptomycin-free and serum-free medium. The plate was then incubated for 12 h, and imaged at a fixed position under an inverted microscope. After incubation for 24 h, they were again imaged at the same position for further analysis.

### Statistical analysis

All data were analyzed using GraphPad Prism 8.0 (GraphPad Software, United States) with Student's *t*-test and One-way Analysis of Variance (One-way ANOVA) applied to compared groups. Data are presented as the mean ± SEM. A *p* value less than 0.05 was considered statistically significant. To analyze survival data, the Kaplan–Meier (K-M) method along with a log-rank test were applied, with significance defined at *p* < 0.05.

## Results

### HCC patients exhibited C1orf122 upregulation and unfavorable survival outcomes

**Figure 1:**
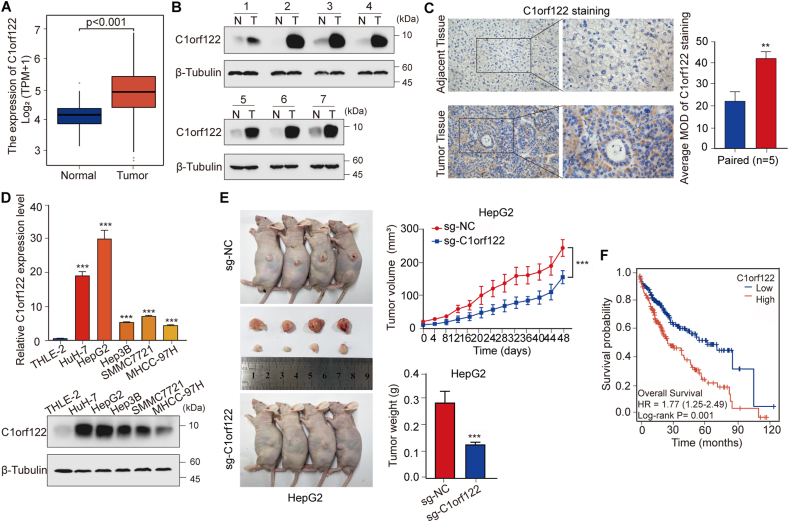
C1orf122 was upregulated in HCC patients and correlated with poor survival outcome. **(A)** The abundance of C1orf122 in HCC and normal tissues was assessed using data from TCGA. **(B)** C1orf122 protein expression in seven pairs of tumor tissues (T) and matching non-cancerous tissues (N) as determined by Western blotting. **(C)** Immunohistochemistry labelling was performed to assess C1orf122 expression in HCC tissues and adjacent tissues. **(D)** qRT-PCR and Western blot experiments were performed to measure the levels of C1orf122 in THLE-2 cells and five HCC cell lines. **(E)** Nude mice were administered and the subcutaneous implants of HepG2 cells were infected with either sg-Control or sg-C1orf122. On day 48 post-implantation, the tumors were collected and photographed, and the tumor growth was tracked. Data (tumor weight and volume data) are expressed as the mean ± SD (*n* = 4). **(F)** The association of C1orf122 levels with the overall survival of patients with HCC was determined by the Kaplan–Meier survival analysis on the GEPIA database. ∗∗*p* < 0.01, ∗∗∗*p* < 0.001.

A pan-cancer analysis was performed using the TCGA database to determine the differential expression of C1orf122 in HCC tissues and normal tissues.^14^ The results indicated that the HCC tissues exhibited a much higher C1orf122 expression than normal tissues (Fig. 1A; Fig. S1A). To validate these findings, we collected and analyzed twelve pairs of tumor and adjacent non-tumor tissues from HCC patients admitted at Bishan Hospital, which is affiliated to Chongqing Medical University. Specifically, we performed WB analysis on seven of these tissue pairings and found that C1orf122 protein levels were significantly higher in tumor tissues than in adjacent non-tumor tissues (Fig. 1B; Fig. S6A). Notably, Immunohistochemistry analysis of the remaining five tissue pairs yielded similar results (Fig. 1C). Furthermore, results of the qRT-PCR and WB analyses showed that the C1orf122 mRNA and protein levels in five HCC cell strains (HuH-7, HepG2, Hep3B, SMMC7721, MHCC-97H) were significantly higher than in normal hepatocytes (THLE-2) (Fig. 1D; Fig. S6B). Additionally, we employed CRISPR-Cas9 to generate C1orf122 knockout HepG2 cell lines, which were then subjected to CDX experiments. This demonstrated that the subcutaneous tumor weights and volumes were much lower in the knockout group than in the controls (Fig. 1E). In the survival analysis using the GEPIA online platform, we found that patients with C1orf122 overexpression had a worse overall survival ratio than those with C1orf122 downregulation (*p* = 0.001) (Fig. 1F). Moreover, Univariate and Multivariate COX proportional hazards analysis revealed that tumor stage (T and M stages) and C1orf122 expression correlated with the overall survival of HCC patients (*p* < 0.05) (Table S1 and Fig. S1B and S1C). Overall, compared to normal tissues and cells, the HCC tumor cells and tissues exhibited a significantly higher C1orf122 expression, suggesting that C1orf122 contributed to the development of HCC and may predict the prognosis of HCC patients.

### C1orf122 stimulated HCC cell growth *in vitro*

**Figure 2:**
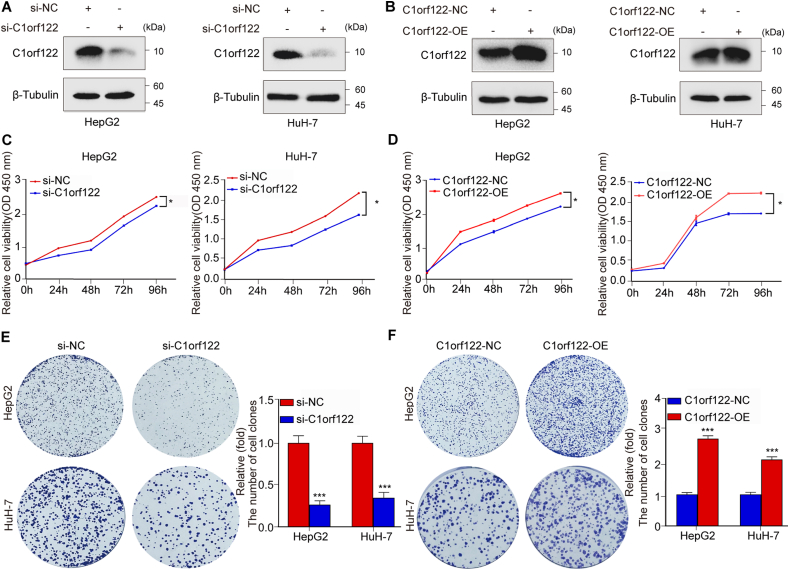
*In vitro*, C1orf122 stimulates HCC cell growth. **(A,B)** The knockdown and overexpression efficiencies of C1orf122 were verified through the Western blot assay. **(C,D)** The viability of cells with either C1orf122 knockdown or overexpression was determined using the CCK-8 assay (mean ± SD (*n* = 3)). (**E**, **F)** The colony formation capacity of cells following C1orf122 knockdown or overexpression as examined by the colony-forming assay (mean ± SD (*n* = 3)). ∗*p* < 0.05, ∗∗∗*p* < 0.001.

To establish the physiological significance of C1orf122 in HCC, we examined the HepG2 and HuH-7 cell lines with C1orf122 knockdown (Fig. 2A; Fig. S2A and S6C) and overexpression (Fig. 2B; Fig. S2B and S6D). Notably, the CCK-8 assay indicated that the C1orf122 knockdown significantly reduced the viability of HepG2 and HuH-7 cells (Fig. 2C), whereas C1orf122 overexpression promoted HepG2 and HuH-7 cell survival (Fig. 2D). On the other hand, the colony formation experiments revealed that C1orf122 knockdown decreased the proliferation capacity of HepG2 and HuH-7 cells (Fig. 2E), whereas C1orf122 overexpression significantly promoted the proliferation in these cell lines (Fig. 2F). These findings underscored the significance of C1orf122 in HCC onset and progression.

### C1orf122 stimulated HCC cell motility and suppressed apoptosis *in vitro*

**Figure 3:**
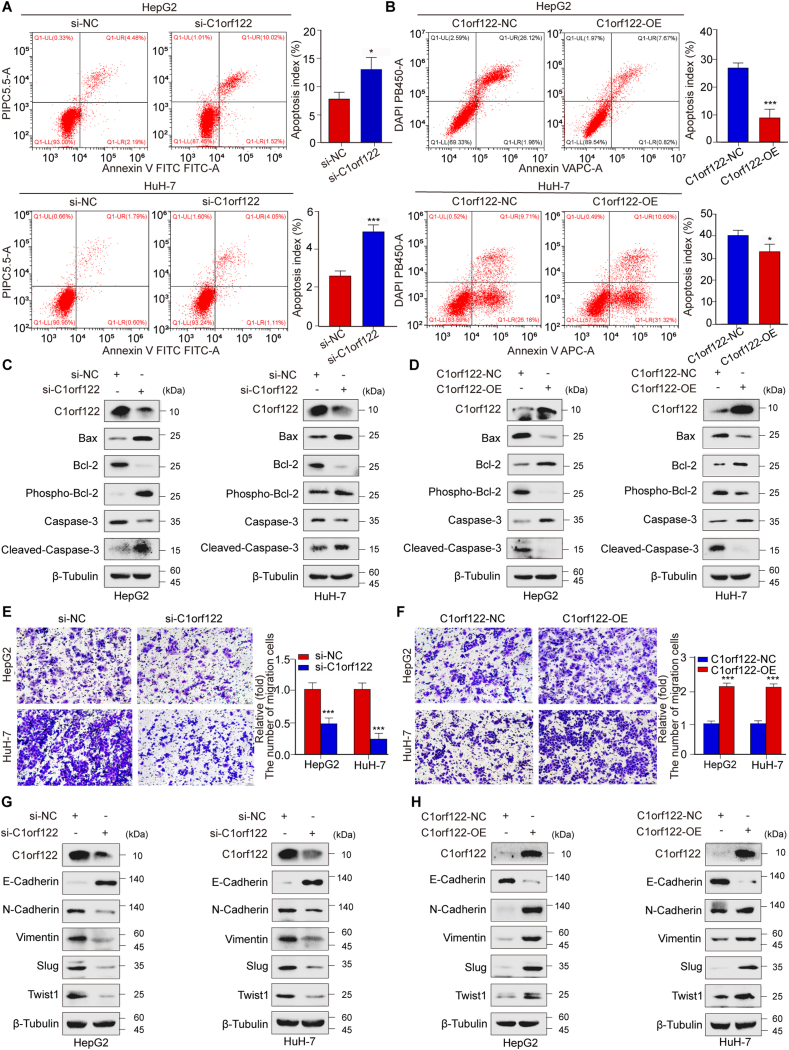
*In vitro*, C1orf122 stimulated HCC cell motility and inhibited apoptosis. **(A**, **B)** Flow cytometry of cell lines with either C1orf122 overexpression or deletion was performed to assess apoptosis rate (mean ± SD (*n* = 3)). **(C**, **D)** Western blot analysis was used to quantify the expression of proteins associated with apoptosis. **(E**, **F)** The migration of C1orf122 knockdown or overexpression cells was determined via Transwell assays (mean ± SD (*n* = 3)). **(G**, **H)** Western blotting was performed to examine the proteins involved in the EMT process. ∗*p* < 0.05, ∗∗*p* < 0.01, ∗∗∗*p* < 0.001.

Further experiments tested the effect of C1orf122 on apoptosis of HCC cell lines. It was observed that C1orf122 knockdown markedly improved the apoptosis rates of HepG2 and HuH-7 cell lines (Fig. 3A). The WB analysis showed that C1orf122 knockdown induced a sharp rise in Bax protein levels, phosphorylated Bcl-2 levels, Cleaved-Caspase-3 levels, and downregulation of total Bcl-2 protein and total Caspase-3 levels, further confirming that C1orf122 knockdown significantly promoted HCC cell apoptosis (Fig. 3C; Fig. S6E and S6F). Conversely, results of flow cytometry (Fig. 3B) and WB (Fig. 3D; Fig. S6G and S6H) revealed that C1orf122 overexpression significantly suppressed the cell apoptosis rates. Furthermore, the Transwell (Fig. 3E) and wound healing (Fig. S3A) assays revealed that C1orf122 knockdown markedly reduced the migratory capacity of HepG2 and HuH-7 cells. The effect of C1orf122 knockdown on epithelial–mesenchymal transition (EMT) pathway's epithelial indicator E-Cadherin, mesenchymal biomarkers N-Cadherin and Vimentin, and associated transcription variables Slug and Twist1 were investigated using WB. It was observed that C1orf122 knockdown increased E-Cadherin expression, but down-regulated N-Cadherin, Vimentin, Slug, and Twist1 expression (Fig. 3G; Fig. S6I and S6J). To further investigate the effect of C1orf122 on the EMT process, HuH-7 cells were transfected with CMV-GFP-Control or CMV-GFP-C1orf122, and changes in cell morphology and E-Cadherin expression levels were observed via immunofluorescence. The experimental results demonstrated that overexpression of C1orf122 induced a morphological shift in HuH-7 cells from a regular polygonal or round shape to a spindle-like shape, accompanied by a significant decrease in E-Cadherin expression. These findings further confirm the significant role of C1orf122 in EMT (Fig. S3C). In the scratch and Transwell experiments, we observed that C1orf122 knockdown significantly reduced the migratory capacity of HCC cells. Conversely, the Transwell (Fig. 3F) and wound healing (Fig. S3B) assays revealed that C1orf122 overexpression enhanced HCC cell migration, which was consistent with WB results (Fig. 3H; Fig. S6K and S6L). Collectively, these findings suggested that C1orf122 inhibited apoptosis and promoted the migration of HCC cells.

### C1orf122 interacted with SRPK1, leading to the activation of the PI3K/AKT/GSK3β signaling pathway, which contributed to its oncogenic properties

**Figure 4:**
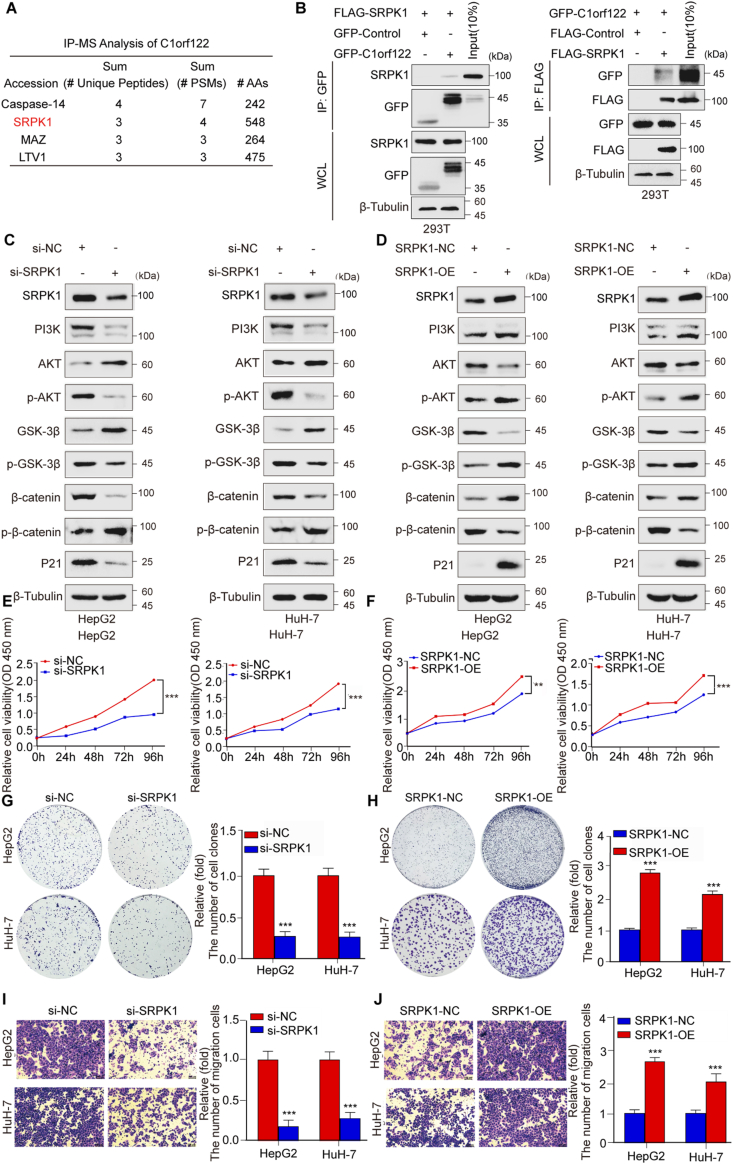
C1orf122 interacted with SRPK1 to activate the PI3K/AKT/GSK3β signaling pathway, inducing oncogenic properties. **(A)** List of four C1orf122-specific binding proteins based on IP-MS analysis. **(B)** In 293T cells, the interactions among GFP-C1orf122 and FLAG-SRPK1 were examined with the Co-IP-WB assay. **(C,D)** The expression levels of proteins associated with the PI3K/AKT/GSK3β signaling pathway were quantified by Western blotting following the knockdown or overexpression of SRPK1. **(E,F)** The CCK-8 assay was conducted to evaluate the viability of cell lines with SRPK1 knockdown or overexpression (mean ± SD (*n* = 3)). **(G,H)** The proliferation of SRPK1 knockdown or overexpressed cell lines was measured by colony formation assay (mean ± SD (*n* = 3)). **(I,J)** The migration of cell lines with altered SRPK1 expression was determined by the Transwell assays (mean ± SD (*n* = 3)). ∗∗*p* < 0.01, ∗∗∗*p* < 0.001.

To further explore the molecular mechanisms by which C1orf122 promoted tumorigenesis in HCC, we examined its interacting partners via IP-MS experiments and Co-IP. Initially, the GFP-Control and GFP-C1orf122 were transfected into 293T cells and performed IP-MS experiments. The results indicated that the intersection of C1orf122 and SRPK1 comprised 252 candidate proteins (Fig. S4A; Fig. 4A and Table S2). Considering the crucial involvement of SRPK1 in cancer pathogenesis, we further explored its mechanisms. Specifically, we investigated the existence of C1orf122-SRPK1 interactions through Co-IP experiments (Fig. 4B). Furthermore, cycloheximide (CHX) chase experiments revealed that knocking down C1orf122 accelerated the degradation of SRPK1 protein (Fig. S4B). Previous research reports that SRPK1 can modulate signaling pathways such as the PI3K/AKT signaling pathway in different malignancies.^15^ Therefore, we hypothesized that SRPK1 may activate the PI3K/AKT pathway and thus, explored its downstream signaling pathways in HCC cells. WB analysis revealed that SRPK1 knockdown in HepG2 and HuH-7 cells significantly downregulated the PI3K protein, reduced AKT protein phosphorylation, and significantly upregulated total AKT protein levels. These phenomena decreased GSK3β phosphorylation and enhanced the total GSK3β protein levels. Consequently, β-catenin was phosphorylated, reducing the total β-catenin content and inhibiting the protein levels of the downstream target gene P21 (Fig. 4C; Fig. S7A and S7B). Moreover, upon SRPK1 overexpression, PI3K was activated, causing AKT protein phosphorylation. The activated AKT protein further phosphorylated GSK3β, leading to its inactivation and β-catenin phosphorylation inhibition, and significant β-catenin aggregation and P21 activation (Fig. 4D; Fig. S7C and S7D). Collectively, these findings demonstrate that SRPK1 significantly increased PI3K/AKT/GSK3β signaling pathway activation and upregulated the protein levels of the downstream target gene P21. Furthermore, we knocked down and overexpressed SRPK1 to better understand its molecular functions in HCC cells. CCK-8 assay results showed that SRPK1 suppression markedly lowered HepG2 and HuH-7 cell viability (Fig. 4E), whereas its overexpression promoted cell viability (Fig. 4F). On the other hand, the colony formation assays revealed that SRPK1 knockdown reduced the proliferation capacity of HepG2 and HuH-7 cells (Fig. 4G), whereas its overexpression significantly increased cell division (Fig. 4H). Furthermore, the Transwell and wound healing tests revealed that SRPK1 knockdown significantly diminished HepG2 and HuH-7 cells’ capacity to migrate (Fig. 4I; Fig. S4B), while its overexpression achieved opposite results (Fig. 4J, Fig. S4C). These findings suggest that SRPK1 regulates HCC cell growth, consistent with the biological function of C1orf122 in these cells. Overall, SRPK1 and C1orf122 promoted the development of HCC, and the C1orf122-SRPK1 interaction stimulated the PI3K/AKT/GSK3β signaling pathway.

### C1orf122 mediated SRPK1 phosphorylation through mTOR kinase

**Figure 5:**
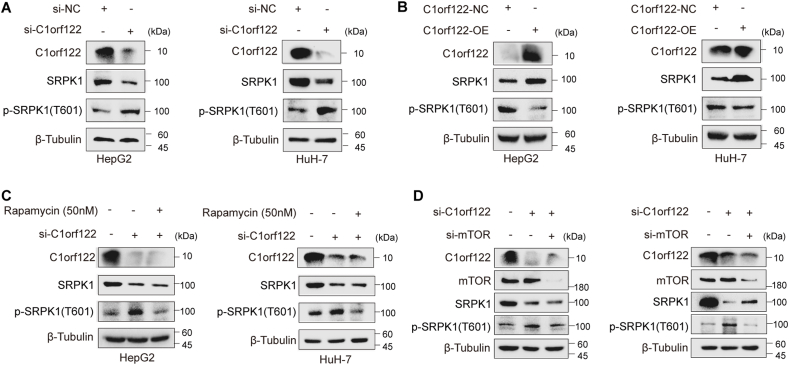
C1orf122 enhanced SRPK1 phosphorylation levels via the mTOR kinase. **(A,B)** Western blot detection of SRPK1 total protein and phosphorylation levels after knockdown or overexpression of C1orf122. **(C)** Western blotting was performed to quantify the expression of SRPK1 protein and phosphorylation after the addition of Rapamycin and knockdown of C1orf122. **(D)** Western blotting was performed to quantify the expression of SRPK1 protein and phosphorylation after the knockdown of mTOR and C1orf122.

To explore the molecular mechanisms underlying C1orf122-SRPK1 interactions, we analyzed the changes in total SRPK1 protein levels and SRPK1 phosphorylation status following C1orf122 knockdown or overexpression in HepG2 and HuH-7 cells. It was observed that HepG2 and HuH-7 cells showed decreased total SRPK1 protein levels following C1orf122 knockdown. This phenomenon was accompanied by a marked increase in SRPK1 phosphorylation at the Thr-601 site (Fig. 5A; Fig. S7E and S7F). On the other hand, although SRPK1's total protein levels increased in tandem with C1orf122 overexpression, the levels of SRPK1 phosphorylation at the Thr-601 site declined significantly (Fig. 5B; Fig. S7G and S7H). To dissect the precise mechanism by which C1orf122 influenced SRPK1 phosphorylation, we predicted the kinases that can regulate SRPK1 phosphorylation at the Thr-601 site using the PhosphoNET Kinase Predictor.^16^ This led to the identification of mTOR as having the highest Kinase Predictor V2 Score. Moreover, adding 50 nM Rapamycin (a classical mTOR inhibitor) significantly reversed the C1orf122 knockdown-induced increase in SRPK1 phosphorylation at the Thr-601 site in HepG2 and HuH-7 cells (Fig. 5C; Fig. S7I and S7J). Meanwhile, the same result can also be obtained by directly knocking down mTOR with siRNA (Fig. 5D; Fig. S7K and S7l). These findings suggest that C1orf122 could modulate SRPK1 phosphorylation via mTOR kinase.

### C1orf122-mediated PI3K/AKT/GSK3β pathway activation was SRPK1-dependent

**Figure 6:**
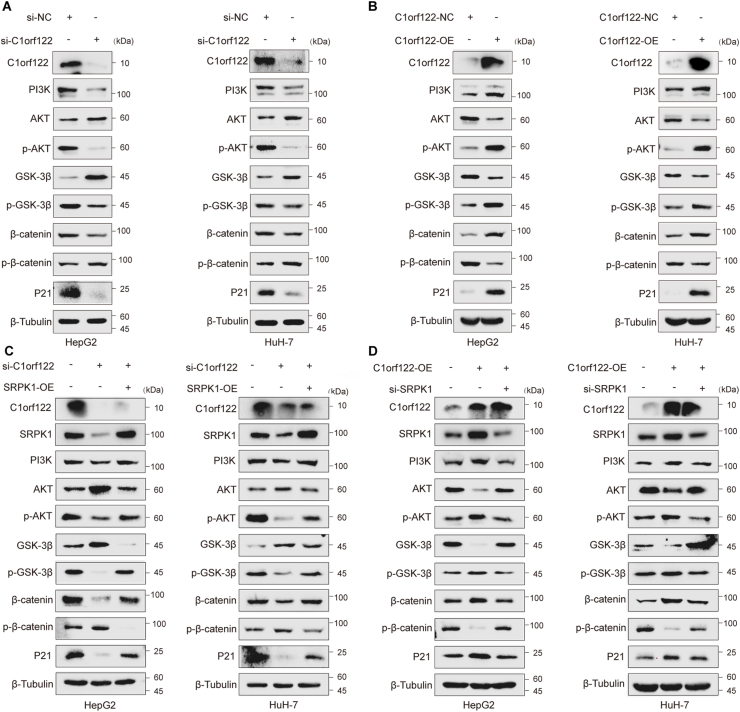
The activation of the PI3K/AKT/GSK3β pathway by C1orf122 was dependent on SRPK1. **(A,B)** The protein levels of markers associated with the PI3K/AKT/GSK3β signaling pathway were analyzed based on Western blotting following knockdown or overexpression of C1orf122. **(C)** The expression of proteins linked to the PI3K/AKT/GSK3β signaling pathway was measured through Western blot analysis following C1orf122 knockdown and subsequent SRPK1 overexpression. **(D)** Western blotting was utilized to quantify the expression of PI3K/AKT/GSK3β signaling-related proteins following C1orf122 overexpression and SRPK1 knockdown.

To further elucidate the mechanisms underlying C1orf122-SRPK1 interactions in HCC, enrichment analysis was performed for HCC and normal tissues on the TCGA database. The Kyoto Encyclopedia of Genes and Genomes (KEGG) enrichment analysis results revealed that C1orf122 was significantly enriched in the PI3K/AKT signaling pathway (Fig. S5), as was SRPK1. Following C1orf122 knockdown in HepG2 and HuH-7 cells, there was a significant decrease in PI3K protein levels, a significant rise in total AKT and GSK3β protein levels, accompanied with a marked decrease in p-AKT and p-GSK3β protein levels. In addition, total β-catenin protein levels were decreased, whereas its phosphorylation levels increased, and P21 protein expression decreased significantly (Fig. 6A; Fig. S8A and S8B). Opposite results were obtained following C1orf122 overexpression (Fig. 6B; Fig. S8C and S8D). The WB analysis results were consistent with the bioinformatics predictions and the SRPK1-regulated signaling pathway experimental results, which showed that C1orf122 could significantly activate the PI3K/AKT/GSK3β signaling pathway. To establish the mechanisms underlying C1orf122-SRPK1 interactions in relation to the activity of the PI3K/AKT signaling pathway, rescue experiments were performed using HepG2 and HuH-7 cells. Initially, C1orf122 was knocked down and SRPK1 was overexpressed. The results indicated that SRPK1 overexpression significantly counteracted the C1orf122 knockdown-induced inhibition of the PI3K/AKT/GSK3β signaling pathway (Fig. 6C; Fig. S8E and S8F). On the other hand, SRPK1 knockdown significantly suppressed the C1orf122 overexpression-induced PI3K/AKT/GSK3β pathway activation (Fig. 6D; Fig. S8G and S8H). Overall, C1orf122 promoted PI3K/AKT/GSK3β signaling pathway activation in HCC cells in a SRPK1-dependent manner.

## Discussion

Analysis of the TCGA database revealed that C1orf122, a previously uncharacterized protein-coding gene, is significantly upregulated in HCC cells, and its expression is associated with poor survival outcomes in HCC patients. Furthermore, both Multivariate and Univariate Cox regression analyses indicated that, in addition to C1orf122 expression, tumor stage is also strongly correlated with overall survival in HCC patients. Due to the tumor's invasive nature, HCC often results in significant differences in clinical outcomes, with many patients experiencing recurrence and metastasis following radical resection surgery. Therefore, identifying novel prognostic biomarkers is crucial for improving risk assessment and tailoring personalized chemotherapy. These findings suggest that C1orf122 could serve as a novel independent prognostic marker for HCC.

Owing to its elevation in both HCC tissues and cells, as well as its potential oncogenic effects in HCC cells, C1orf122 was further investigated through *ex vivo* and *in vivo* functional experiments. The *in vitro* findings revealed that silencing C1orf122 in HepG2 and HuH-7 cells significantly reduced cell proliferation. Conversely, C1orf122 overexpression significantly promoted cell growth. Specifically, C1orf122 promoted HCC cell growth via apoptosis inhibition, as indicated by its significant effect on the expression of apoptotic pathway-related genes such as Bax, Bcl-2, Phospho-Bcl-2, Caspase-3, and Cleaved-Caspase-3, among others. Among these genes, Bax and Bcl-2 belong to the Bcl-2 family involved in the regulation of mitochondrial membrane permeability, thereby modulating apoptotic process. The Bax protein is particularly essential in the apoptosis initiation process, primarily serving as a pro-apoptotic protein. It increases mitochondrial membrane permeability, causing the release of cytochrome C and other apoptosis-related substances into the cytoplasm.^17^ Conversely, Bcl-2 functions as an anti-apoptotic protein, preventing cell apoptosis by suppressing the activity of pro-apoptotic factors, such as Bax.^18^ Furthermore, Caspase-3, a member of the Caspase enzyme family, functions as an apoptosis executor via promoting cellular protein degradation.^19^ Our findings also revealed that C1orf122 knockdown strongly reduced the migratory capabilities of HepG2 and HuH-7 cell lines, while its overexpression exerted opposite effects, promoting cell migration. Moreover, C1orf122 was significantly involved in EMT activation. In the epithelial cells, E-cadherin, a type I transmembrane protein, plays a crucial role in cell adhesion, migration, and proliferation. Therefore, we infer that E-Cadherin downregulation is one of the hallmark steps in both EMT and cancer metastases.^20^ Furthermore, N-Cadherin, a single-chain transmembrane glycoprotein, is known to promote cell adhesion in a process that is calcium-dependent. Consequently, N-Cadherin upregulation has been linked to enhanced invasion and metastasis of malignant tumors.^21^ Additionally, vimentin, a type III intermediate that is upregulated during EMT, can influence cell shape and motility, thus promoting EMT.^22^ Transcription Factors (TFs) such as Slug and Twist1 have been shown to stimulate the EMT process.^23^ Specifically, Slug binds to conserved E-Boxes, suppressing the expression of genes, such as E-cadherin and tight junction proteins.^24^ On the other hand, Twist1, a highly conserved TF, binds to E-box sequences, downregulating E-Cadherin and activating mesenchymal cell markers, thus promoting EMT.^25^ Overall, our findings revealed that C1orf122 exerted oncogenic effects in HCC by enhancing cell proliferation, suppressing apoptosis, and facilitating cell migration. Given the crucial involvement of C1orf122 in HCC development, we further evaluated its therapeutic value. It was observed that C1orf122 knockdown in HepG2 cells significantly decreased the growth of subcutaneous xenograft tumors, suggesting that C1orf122 may be a viable therapeutic target for HCC treatment.

To elucidate the molecular mechanisms underlying C1orf122's role in HCC, we first identified its interacting protein SRPK1 via IP-MS. Notably, the Co-IP assays confirmed that C1orf122 interacted with SRPK1. Studies have shown that SRPK1 exhibits oncogenic effects in various tumor types and regulates the PI3K/AKT signaling pathway.^26^ It can also activate the AKT-mediated NF-κB pathway, to exert anti-apoptotic processes.^27^ Moreover, SRPK1 participates in the EGF-induced EGFR activation,^28^ interacts with AKT, and regulates AKT phosphorylation.^29^ Moreover, SRPK1 directly regulates the Wnt/β-catenin and JAK2/STAT-3 signaling pathways, thus promoting glioma development.^8^ Aberrant SRPK1 expression can promote the Wnt/β-catenin pathway activation, a phenomenon that induce stem cell phenotypes in NSCLC cells.^30^ Notably, the phosphorylation of Ser9, which is located on the GSK3β kinase inhibitory protein, can activate the Wnt/β-catenin pathway and phosphorylate β-catenin 31. The PI3K/AKT pathway also primarily targets it, with GSK3β Ser9 potentially undergoing phosphorylation following AKT activation.^32^ Moreover, the non-kinase structural domain of SRPK1 can facilitate its interaction with GSK3β, promoting GSK3β autophosphorylation at the Ser9 site, thus enhancing gefitinib resistance in NSCLC.^27^ Collectively, these results suggest that SRPK1 can regulate multiple signaling pathways, leading to β-catenin accumulation in the nucleus and activating the Wnt pathway. Data indicates that SRPK1 can mediate the communication between EGFR and Wnt/β-catenin upstream signals and their downstream effectors, such as AKT. Moreover, SRPK1 interacts directly with GSK3β, further highlighting its significance in PI3K/AKT pathways and downstream signaling. Our *in vitro* functional experiments confirmed that SRPK1 significantly enhanced HCC cell growth and expansion. Furthermore, consistent with existing literature, our WB analysis confirmed that SRPK1 exerted oncogenic effects via activating the PI3K/AKT/GSK3β signaling pathway in HCC cells. The PI3K/AKT/GSK3β signaling pathway is a frequently dysregulated signaling cascade in various human tumor types.^33^ This pathway primarily stimulates the receptor tyrosine kinases or G protein-coupled receptors, triggering the PI3K recruitment and activation, resulting in PIP3 production.^34^ This process, in turn, attracts AKT towards the cell membrane, where PDK1 and mTORC2 phosphorylate and activate it, thereby regulating various downstream effectors.^35^^,^^36^ Meanwhile, AKT phosphorylation decreases the activity of GSK3β, one of the downstream targets of AKT. Overall, there is increasing research evidence that the PI3K/AKT/GSK3β signaling pathway is involved in HCC onset and progression.^37^

In this study, WB analysis results confirmed that C1orf122 overexpression increased SRPK1 protein levels and decreased SRPK1 phosphorylation at the Thr601 site. According to previous research, SRPK1, a crucial protein kinase, primarily phosphorylates SR proteins to influence RNA splicing mechanism.^38^ Nonetheless, the phosphorylation state of SRPK1 may also regulate its activity and function, a process that involves upstream kinases that can phosphorylate SRPK1. Notably, AKT was found to induce SRPK1 phosphorylation by acting as a kinase at its T326 and S587 sites.^39^ Herein, we searched the PhosphoNET Kinase Predictor and identified mTOR as the kinase affecting SRPK1 phosphorylation at the Thr601 site. Subsequently, we added the mTOR inhibitor Rapamycin to C1orf122-overexpressing cells, which revealed that C1orf122 mediated the SRPK1 phosphorylation at the Thr601 site via mTOR. Enrichment analysis of the TCGA dataset and the SRPK1-regulated signaling pathway, along with WB analyses, further revealed that C1orf122 markedly stimulated and activated the PI3K/AKT/GSK3β signaling pathway in HCC cells. Additional rescue assays confirmed that this activation was SRPK1-dependent.

## Conclusions

**Figure 7:**
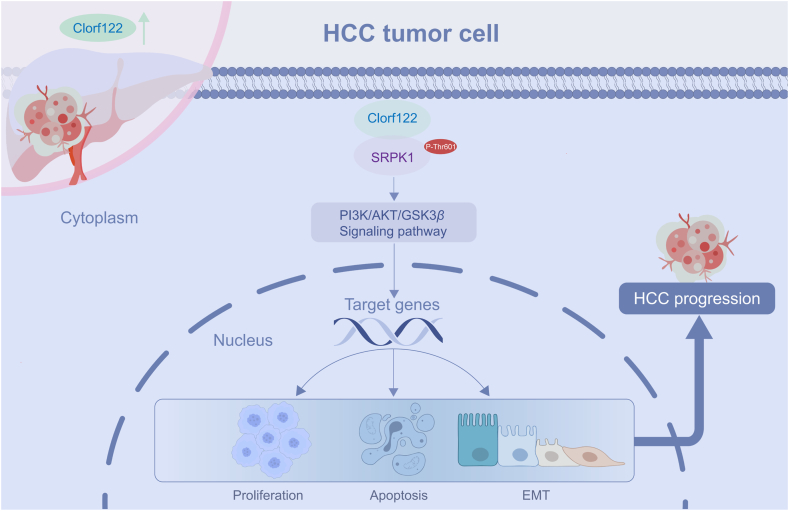
A proposed model explaining the biological function of C1orf122 in the progression of HCC. C1orf122 binds to SRPK1 and mediates SRPK1 phosphorylation at the Thr601 site. This activates the PI3K/AKT/GSK3β via SRPK1, ultimately promoting the progression of HCC.

The present findings revealed significant C1orf122 overexpression in HCC, which correlated with a poor prognosis. Moreover, C1orf122 was found to be an oncogenic factor in HCC, promoting HCC cell proliferation and migration while inhibiting apoptosis. Mechanistically, C1orf122 bound to SRPK1 and mediated SRPK1 phosphorylation at the Thr601 site via mTOR kinase. Moreover, C1orf122 activated the PI3K/AKT/GSK3β pathway via SRPK1. These findings demonstrate the role of C1orf122 in HCC development via the SRPK1-PI3K/AKT/GSK3β axis, highlighting its potential as a diagnostic or therapeutic target for HCC (Fig. 7).

## CRediT authorship contribution statement

**Jing Cai:** Writing – original draft, Methodology, Investigation, Data curation, Conceptualization. **Li Rong:** Writing – review & editing, Funding acquisition. **Runzhi Wang:** Project administration, Methodology. **Zaikuan Zhang:** Resources. **Haiming Sun:** Resources. **Juan Chen:** Resources. **Dunchu Weng:** Resources. **Xinyi Li:** Supervision. **Xiaosong Feng:** Supervision. **Peiyi Lin:** Project administration. **Shengming Xu:** Project administration. **Zhihong Jiang:** Project administration. **Yajun Xie:** Writing – review & editing, Project administration, Data curation, Conceptualization. **Qin Zhou:** Funding acquisition, Conceptualization.

## Data availability

The datasets used and/or analysed during the current study are available from the corresponding author on reasonable request. The data related to HCC patients required for the bioinformatics analysis in this study was downloaded from the TCGA database (https://www.cancer.gov/ccg/research/genome-sequencing/tcga). We predicted the kinases that can regulate SRPK1 phosphorylation at the Thr-601 site using the PhosphoNET Kinase Predictor (http://www.phosphonet.ca/kinasepredictor.aspx?uni=Q96SB4&ps=T601).

## Ethics approval and consent to participate

All animal experiments were performed in accordance with the National Institute of Health's Guide for the Care and Use of Laboratory Animals and were approved by the Ethics Committee of Chongqing Medical University in China (IACUC-CQMU-2024-0102). Additionally, the HCC tissues were obtained from Bishan Hospital of Chongqing Medical University. All patients have signed informed consent, and it complied with the principles of medical ethics and the Declaration of Helsinki, which was approved by the Ethics Committee of Bishan Hospital of Chongqing Medical University (cqbykll-20240928-01).

## Funding

This work was supported by the key project of the 10.13039/501100001809National Natural Science Foundation of China (No. 82030065); the National Key R&D Program of China (No. 2023YFA1801900); and the Bishan District Science and Technology Bureau Project of Chongqing (No. BSKJ2024006).

## Conflict of interests

The authors state that they possess no competing interests.

## References

[1] Sung H, Ferlay J, Siegel RL (2021). Global cancer statistics 2020: GLOBOCAN estimates of incidence and mortality worldwide for 36 cancers in 185 countries. CA Cancer J Clin.

[2] Feng M, Pan Y, Kong R, Shu S (2020). Therapy of primary liver cancer. Innovation.

[3] Chen W, Chiang CL, Dawson LA (2021). Efficacy and safety of radiotherapy for primary liver cancer. Chin Clin Oncol.

[4] Donne R, Lujambio A (2023). The liver cancer immune microenvironment: therapeutic implications for hepatocellular carcinoma. Hepatology.

[5] Wang Y, Deng B. (2023). Hepatocellular carcinoma: molecular mechanism, targeted therapy, and biomarkers. Cancer Metastasis Rev.

[6] McGlynn KA, Petrick JL, El-Serag HB. (2021). Epidemiology of hepatocellular carcinoma. Hepatology.

[7] Conci S, Cipriani F, Donadon M (2022). Hepatectomy for metabolic associated fatty liver disease (MAFLD) related HCC: propensity case-matched analysis with viral- and alcohol-related HCC. Eur J Surg Oncol.

[8] Shi M, Sun D, Deng L, Liu J, Zhang MJ (2024). SRPK1 promotes glioma proliferation, migration, and invasion through activation of Wnt/β-Catenin and JAK-2/STAT-3 signaling pathways. Biomedicines.

[9] Seitz HK, Bataller R, Cortez-Pinto H (2018). Alcoholic liver disease. Nat Rev Dis Primers.

[10] Xu X, Wei Y, Wang S, Luo M, Zeng H (2017). Serine-arginine protein kinase 1 (SRPK1) is elevated in gastric cancer and plays oncogenic functions. Oncotarget.

[11] Gout S, Brambilla E, Boudria A (2012). Abnormal expression of the pre-mRNA splicing regulators SRSF1, SRSF2, SRPK1 and SRPK2 in non small cell lung carcinoma. PLoS One.

[12] Ren G, Sheng L, Liu H, Sun Y, An Y, Li Y (2015). The crucial role of SRPK1 in TGF-β-induced proliferation and apoptosis in the esophageal squamous cell carcinomas. Med Oncol.

[13] Hayes GM, Carrigan PE, Miller LJ. (2007). Serine-arginine protein kinase 1 overexpression is associated with tumorigenic imbalance in mitogen-activated protein kinase pathways in breast, colonic, and pancreatic carcinomas. Cancer Res.

[14] The cancer genome atlas database. https://www.cancer.gov/ccg/research/genome-sequencing/tcga.

[15] Duggan WP, O’Connell E, Prehn JHM, Burke JP (2022). Serine-arginine protein kinase 1 (SRPK1): a systematic review of its multimodal role in oncogenesis. Mol Cell Biochem.

[16] PhosphoNET. http://www.phosphonet.ca/kinasepredictor.aspx?uni=Q96SB4&ps=T601.

[17] Lalier L, Cartron PF, Juin P (2007). Bax activation and mitochondrial insertion during apoptosis. Apoptosis.

[18] Czabotar PE, Garcia-Saez AJ. (2023). Mechanisms of BCL-2 family proteins in mitochondrial apoptosis. Nat Rev Mol Cell Biol.

[19] Porter AG, Jänicke RU. (1999). Emerging roles of caspase-3 in apoptosis. Cell Death Differ.

[20] Serrano-Gomez SJ, Maziveyi M, Alahari SK. (2016). Regulation of epithelial-mesenchymal transition through epigenetic and post-translational modifications. Mol Cancer.

[21] Mrozik KM, Blaschuk OW, Cheong CM, Zannettino ACW, Vandyke K (2018). N-cadherin in cancer metastasis, its emerging role in haematological malignancies and potential as a therapeutic target in cancer. BMC Cancer.

[22] Paulin D, Lilienbaum A, Kardjian S, Agbulut O, Li Z. Vimentin: regulation and pathogenesis. Biochimie. 2022;197:96–112. 10.1016/j.biochi.2022.02.003 35151830

[23] Bustamante A, Baritaki S, Zaravinos A, Bonavida B (2024). Relationship of signaling pathways between RKIP expression and the inhibition of EMT-inducing transcription factors SNAIL1/2, TWIST1/2 and ZEB1/2. Cancers (Basel).

[24] Assani G, Zhou Y. (2019). Effect of modulation of epithelial-mesenchymal transition regulators Snail 1 and Snail2 on cancer cell radiosensitivity by targeting of the cell cycle, cell apoptosis and cell migration/invasion. Oncol Lett.

[25] Seo J, Ha J, Kang E, Cho S (2021). The role of epithelial-mesenchymal transition-regulating transcription factors in anti-cancer drug resistance. Arch Pharm Res (Seoul).

[26] Guo W, Hu Z. (2023). SRPK1 promotes sepsis-induced acute lung injury via regulating PI3K/AKT/FOXO3 signaling. Immunopharmacol Immunotoxicol.

[27] Huang JQ, Li HF, Zhu J (2021). SRPK1/AKT axis promotes oxaliplatin-induced anti-apoptosis via NF-κB activation in colon cancer. J Transl Med.

[28] Kurimchak AM, Kumar V, Herrera-Montávez C (2020). Kinome profiling of primary endometrial tumors using multiplexed inhibitor beads and mass spectrometry identifies SRPK1 as candidate therapeutic target. Mol Cell Proteomics.

[29] Wang P, Zhou Z, Hu A (2014). Both decreased and increased SRPK1 levels promote cancer by interfering with PHLPP-mediated dephosphorylation of Akt. Mol Cell.

[30] Gong L, Song J, Lin X (2016). Serine-arginine protein kinase 1 promotes a cancer stem cell-like phenotype through activation of Wnt/β-catenin signalling in NSCLC. J Pathol.

[31] Anand AA, Khan M, V M, Kar D (2023). The molecular basis of Wnt/β-Catenin signaling pathways in neurodegenerative diseases. Int J Cell Biol.

[32] Yuan Y, Fan Y, Gao Z (2020). SHP2 promotes proliferation of breast cancer cells through regulating cyclin D1 stability via the PI3K/AKT/GSK3β signaling pathway. Cancer Biol Med.

[33] He Y, Sun MM, Zhang GG (2021). Targeting PI3K/Akt signal transduction for cancer therapy. Signal Transduct Targeted Ther.

[34] Qiao M, Sheng S, Pardee AB. (2008). Metastasis and AKT activation. Cell Cycle.

[35] Dieterle AM, Böhler P, Keppeler H (2014). PDK1 controls upstream PI3K expression and PIP3 generation. Oncogene.

[36] Jhanwar-Uniyal M, Wainwright JV, Mohan AL (2019). Diverse signaling mechanisms of mTOR complexes: mTORC1 and mTORC2 in forming a formidable relationship. Adv Biol Regul.

[37] Bai C, Zhao J, Su J (2022). Curcumin induces mitochondrial apoptosis in human hepatoma cells through BCLAF1-mediated modulation of PI3K/AKT/GSK-3β signaling. Life Sci.

[38] Gou LT, Lim DH, Ma W (2020). Initiation of parental genome reprogramming in fertilized oocyte by splicing kinase SRPK1-Catalyzed protamine phosphorylation. Cell.

[39] Zhou Z, Qiu J, Liu W (2018). The Akt-SRPK-SR axis constitutes a major pathway in transducing EGF signaling to regulate alternative splicing in the nucleus. Mol Cell.

