# Supplementary material for: Identifying C1orf122 as a potential HCC exacerbated biomarker dependently of SRPK1 regulates PI3K/AKT/GSK3β signaling pathway
Source: Genes Dis. 2025 Jun 18;13(1):101721. doi: 10.1016/j.gendis.2025.101721 (PMC12552961; doi:10.1016/j.gendis.2025.101721)
Supplement: Multimedia component 1 [file mmc1.docx]

**Supplementary information**

**Table S1** Correlation between C1of122 expression and clinicopathological characteristics of HCC.

| Characteristics | Total (N) | Univariate analysis | |  | Multivariate analysis | |
| --- | --- | --- | --- | --- | --- | --- |
|  |  | Hazard ratio (95% CI) | P value |  | Hazard ratio (95% CI) | P value |
| C1orf122 | 373 |  |  |  |  |  |
| Low | 87 | Reference |  |  | Reference |  |
| High | 186 | 1.788 (1.256 - 2.546) | **0.001** |  | 1.788 (1.226 - 2.606) | **0.003** |
| Pathologic T stage | 370 |  |  |  |  |  |
| T1 | 183 | Reference |  |  | Reference |  |
| T2 | 94 | 1.431 (0.902 - 2.268) | 0.128 |  | 1.333 (0.817 - 2.176) | 0.250 |
| T3 | 80 | 2.674 (1.761 - 4.060) | **< 0.001** |  | 1.935 (0.257 - 14.554) | 0.521 |
| T4 | 13 | 5.386 (2.690 - 10.784) | **< 0.001** |  | 3.219 (0.383 - 27.043) | 0.282 |
| Pathologic N stage | 258 |  |  |  |  |  |
| N0 | 254 | Reference |  |  |  |  |
| N1 | 4 | 2.029 (0.497 - 8.281) | 0.324 |  |  |  |
| Pathologic M stage | 272 |  |  |  |  |  |
| M0 | 268 | Reference |  |  |  |  |
| M1 | 4 | 4.077 (1.281 - 12.973) | **0.017** |  |  |  |
| Pathologic stage | 349 |  |  |  |  |  |
| Stage I&Stage II | 259 | Reference |  |  | Reference |  |
| Stage III&Stage IV | 90 | 2.504 (1.727 - 3.631) | **< 0.001** |  | 1.368 (0.188 - 9.956) | 0.757 |
| Gender | 373 |  |  |  |  |  |
| Female | 121 | Reference |  |  |  |  |
| Male | 252 | 0.793 (0.557 - 1.130) | 0.200 |  |  |  |
| Age | 373 |  |  |  |  |  |
| <= 60 | 177 | Reference |  |  |  |  |
| > 60 | 196 | 1.205 (0.850 - 1.708) | 0.295 |  |  |  |

**Figure S1**


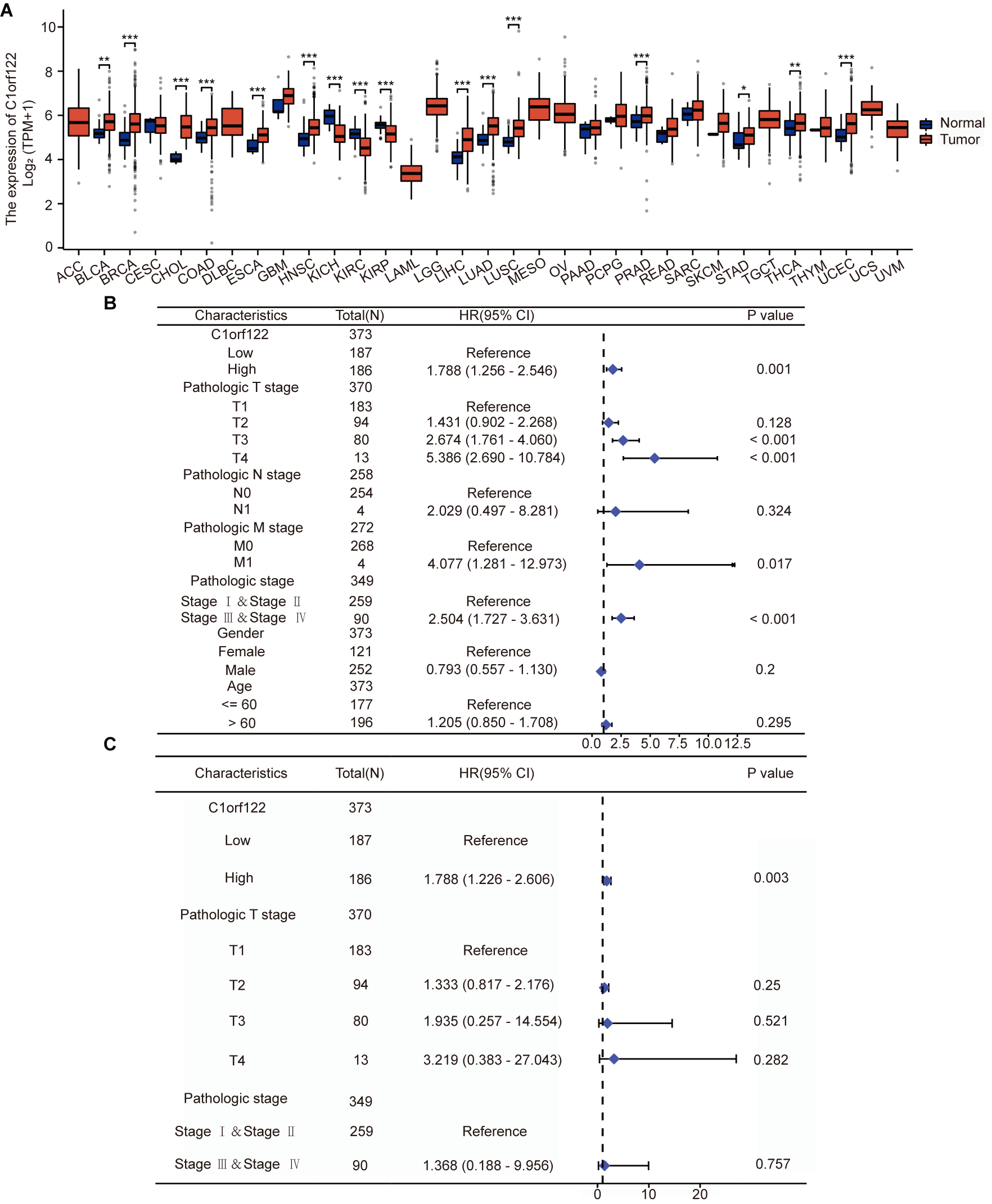


**Figure S1** C1orf122 is highly expressed in HCC and is correlated with clinical stage. (**A**) The expression level of C1orf122 in various tumor tissues and normal tissues was predicted by a pan-cancer analysis in the TCGA database. (**B**) Univariate Cox regression analysis indicated that the expression level of C1orf122 and tumor stage (T and M stage) were correlated with the overall survival of HCC patients. (**C**) Multivariate regression analysis revealed that only the expression level of C1orf122 was significantly correlated with overall survival. **P* < 0.05, ***P* < 0.01, ****P* < 0.001.

**Figure S2**


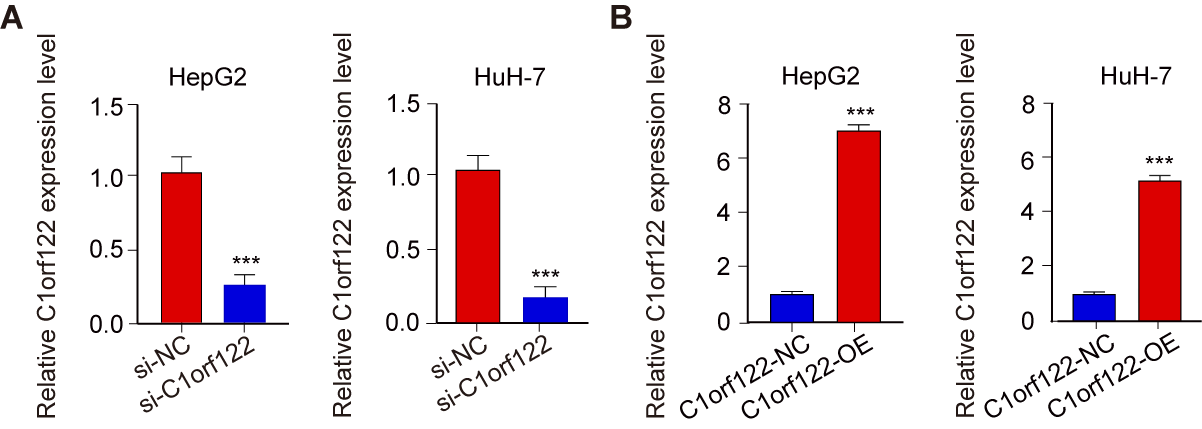


**Figure S2** The knockdown and overexpression efficiency of C1orf122 was verified by qRT–PCR. (**A**, **B**) The mRNA expression levels of C1orf122 in HepG2 and HuH-7 cells were measured by qRT–PCR following either knockdown or overexpression (mean ± SD (*n* = 3)). ****P* < 0.001.

**Figure S3**


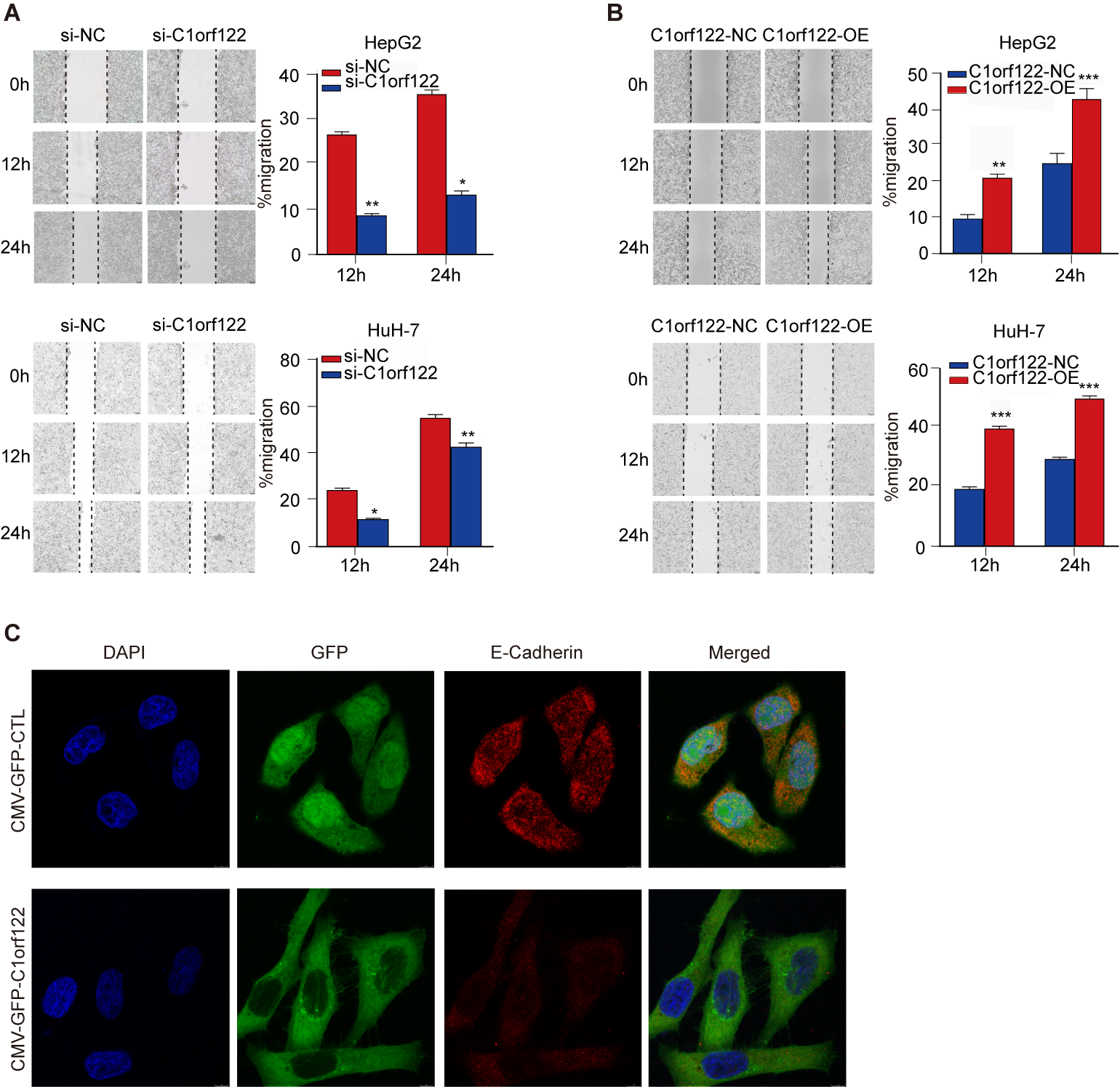


**Figure S3** The migration ability of C1orf122 was verified by wound healing experiments, and the morphology of HCC cells overexpressing C1orf122 was observed by immunofluorescence. (**A**) The migration ability of HepG2 and HuH-7 cells following C1orf122 knockdown was assessed via wound healing assays (mean ± SD (*n* = 3)). (**B**) Wound healing assays were performed to assess the migratory ability of HepG2 and HuH-7 cells after overexpression of C1orf122 (mean ± SD (*n* = 3)). (**C**) After the transfection of CMV-GFP-Control or CMV-GFP-C1orf122 into HuH-7 cells, morphological changes of the cells and changes of E-cadherin fluorescence staining were observed via immunofluorescence. Scale bar, 5 μm. **P* < 0.05, ***P* < 0.01, ****P* < 0.001.

**Figure S4**


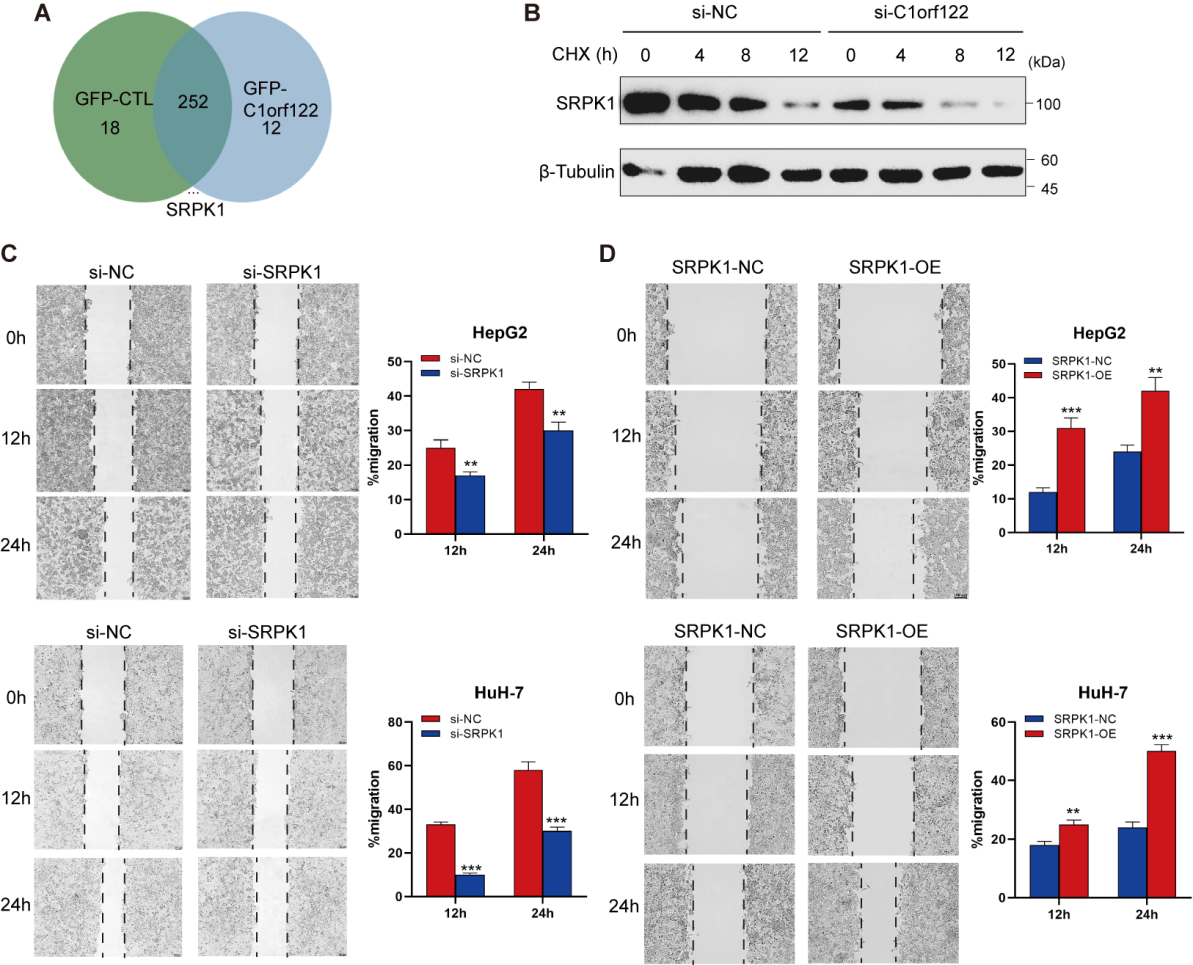


**Figure S4** Venn diagram of the IP/MS and SRPK1 wound healing experiment analysis. (**A**) Venn diagram results indicated the interacting proteins of C1orf122 predicted by IP/MS. (**B**) After HuH-7 cells were knocked down with si-C1orf122 and treated with 100 μg/mL cycloheximide (CHX), the half-life of the SRPK1 protein was detected via western blotting. (**C**) The migration ability of HepG2 and HuH-7 cells after SRPK1 knockdown was assessed via wound healing assays (mean ± SD (*n* = 3)). (**D**) Wound healing assays were performed to assess the migratory ability of HepG2 and HuH-7 cells after overexpression of SRPK1 (mean ± SD (*n* = 3)). ***P* < 0.01, ****P* < 0.001.

**Figure S5**


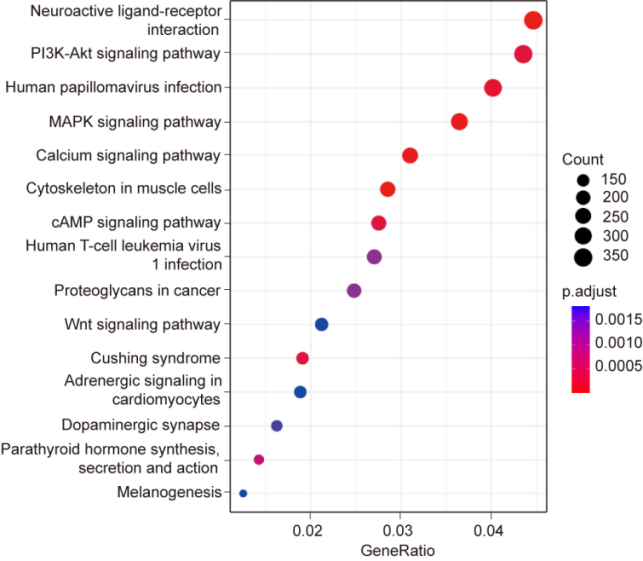


**Figure S5** KEGG analysis results predicting the signaling pathways. The KEGG analysis results showed that enrichment analysis of the HCC and adjacent tissue datasets from the TCGA database predicted the signaling pathways regulated by C1orf122.

**Figure S6**


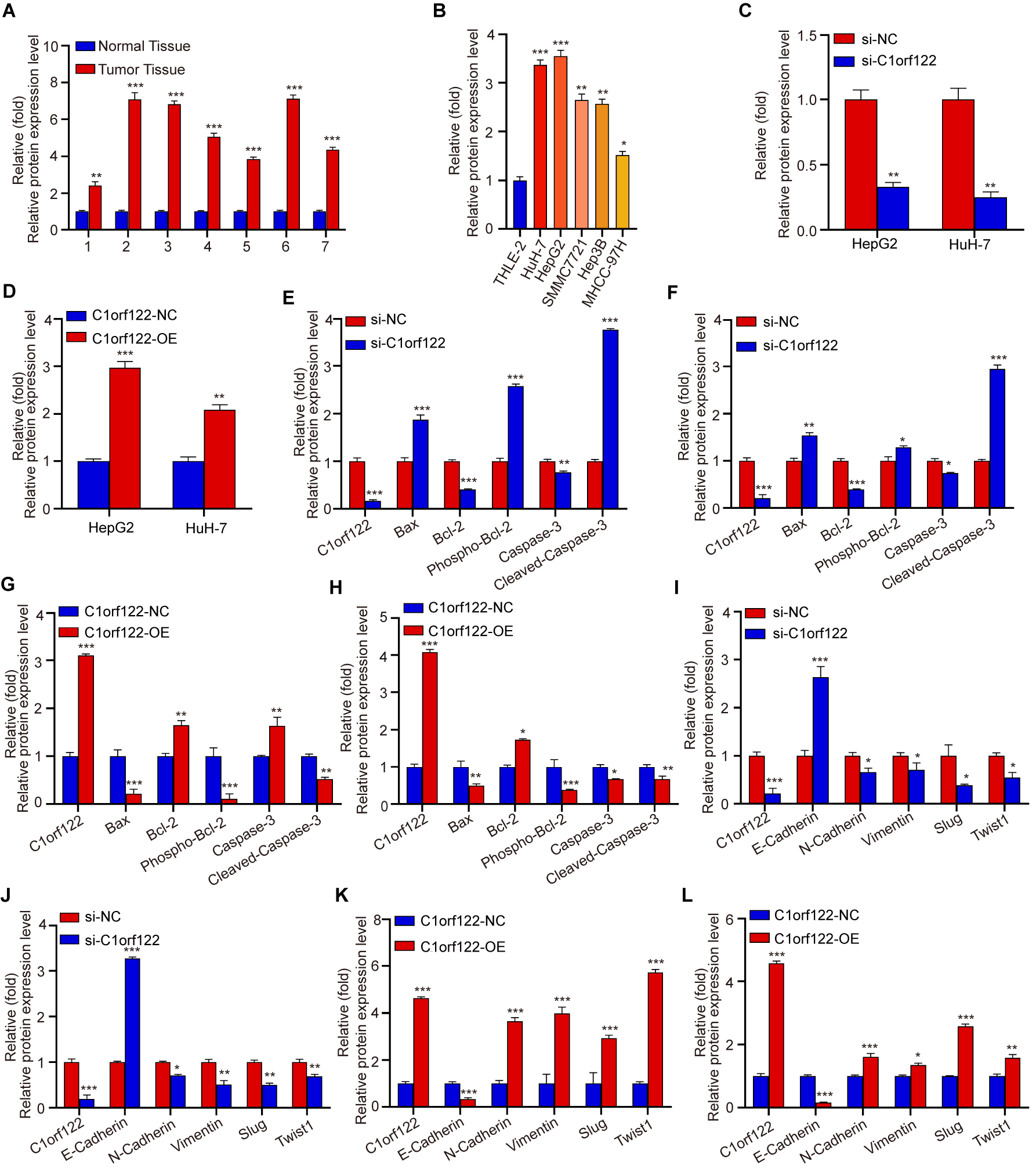


**Figure S6** Densitometric analysis of the protein expression levels shown in Figure 1, Figure 2, and Figure 3. The expression levels of all target proteins were normalized to those of β-Tubulin. All the bar graphs were arranged in sequential order (mean ± SD (*n* = 3)). **P* < 0.05, ***P* < 0.01, ****P* < 0.001.

**Figure S7**

**
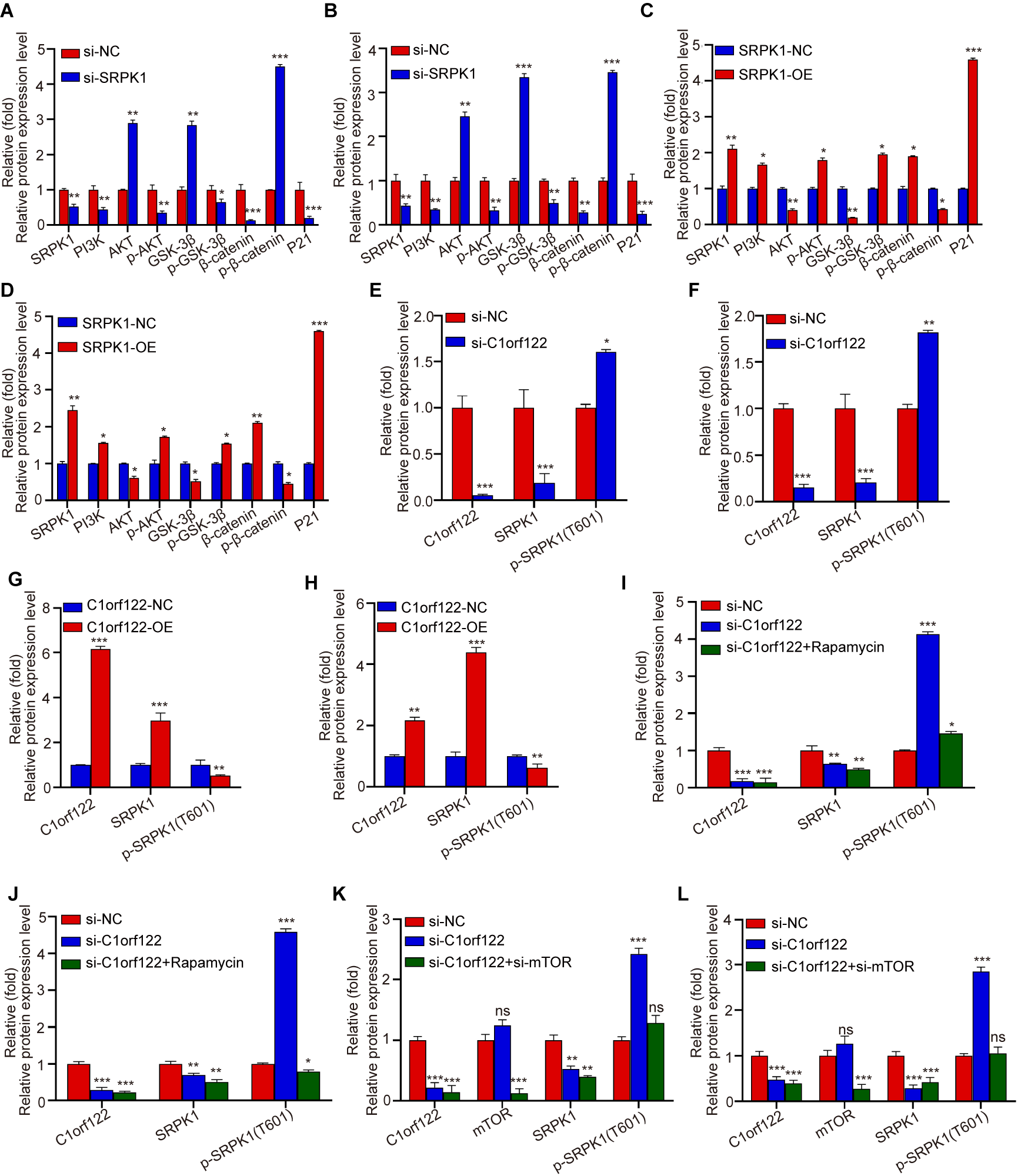
**

**Figure S7** Densitometric analysis of the protein expression levels in Figure 4 and Figure 5. The expression levels of all target proteins were normalized to those of β-Tubulin. All the bar graphs were arranged in sequential order (mean ± SD (*n* = 3)). **P* < 0.05, ***P* < 0.01, ****P* < 0.001.

**Figure S8**

**
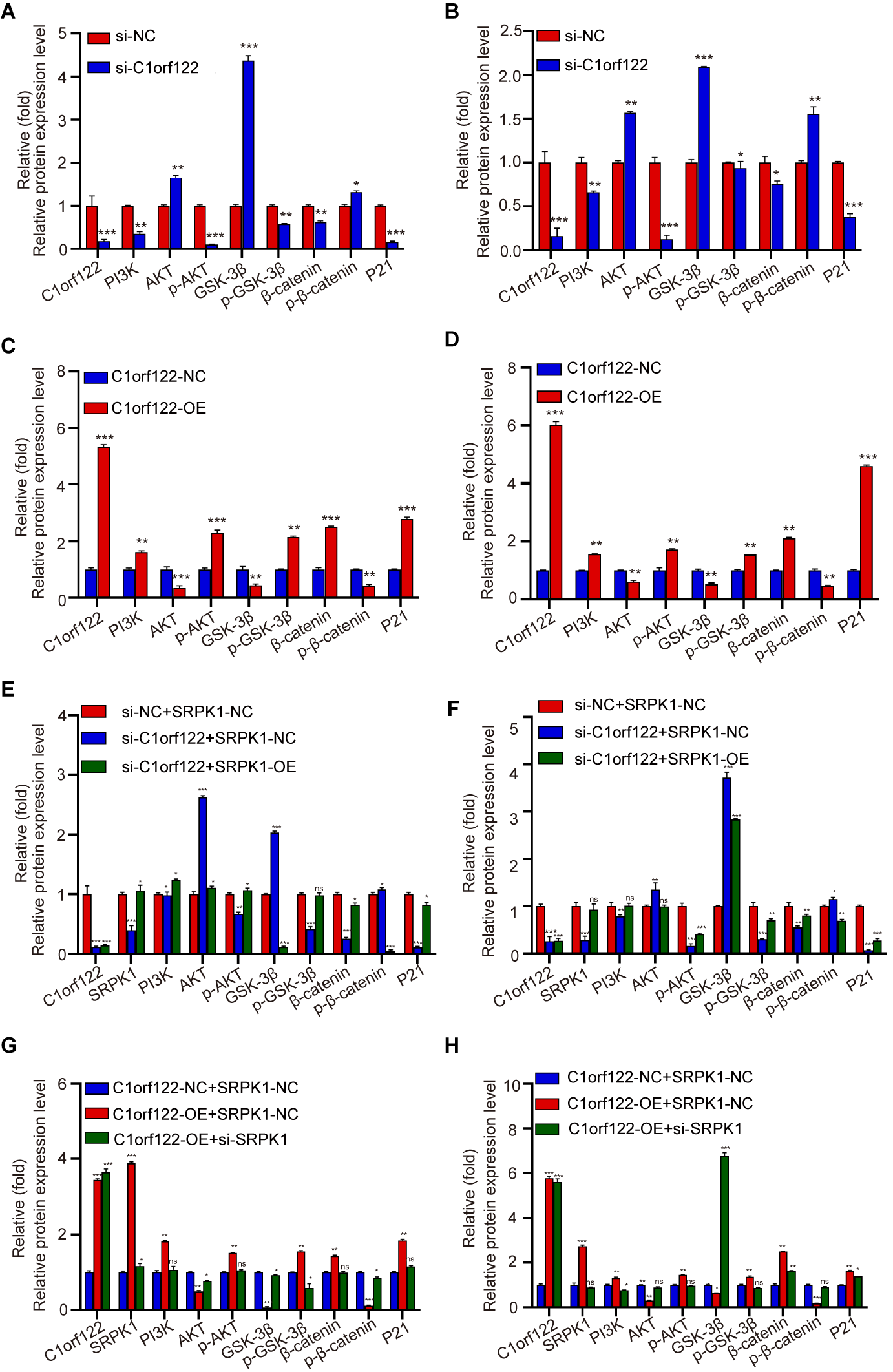
**

**Figure S8** Densitometric analysis of protein expression levels in Figure 6. The expression levels of all target proteins were normalized to those of β-Tubulin. All bar graphs were arranged in sequential order (mean ± SD (*n* = 3)). **P* < 0.05, ***P* < 0.01, ****P* < 0.001.
